# The Triglyceride Glucose Index Is a Risk Factor for Enlarged Perivascular Space

**DOI:** 10.3389/fneur.2022.782286

**Published:** 2022-02-04

**Authors:** Yazhen Cai, Binxiong Chen, Xiaoyi Zeng, Meirong Xie, Xiaolan Wei, Jiangping Cai

**Affiliations:** Department of Neurology, The First Hospital of Quanzhou Affiliated to Fujian Medical University, Quanzhou, China

**Keywords:** triglyceride, glucose, insulin resistance, enlarged perivascular space, non-diabetic population

## Abstract

The triglyceride glucose (TyG) index is considered a simple surrogate marker for insulin resistance and has been associated with cerebrovascular diseases. However, limited information is available regarding its association with the subclinical cerebral small vessel disease (CSVD). Here, we investigated the association of TyG index with the burden and distribution of enlarged perivascular space (EPVS) in the non-diabetic population. The data of 531 non-diabetic patients from 2017 to 2020 were assessed. Participants were grouped according to the burden of EPVS. TyG index was calculated using the log scale of fasting triglycerides (mg/dl) × fasting glucose (mg/dl)/2. The association of TyG index with EPVS burden and distribution was evaluated. In the multivariable logistic regression analysis, the TyG index was associated with moderate to severe EPVS [odds ratio (OR): 2.077; 95% *CI* = 1.268–3.403]. The TyG index was significantly associated with an increased risk of moderate to severe EPVS in subgroups of age <65 years, male, diastolic blood pressure (DBP) <90 mmHg, low-density lipoprotein cholesterol (LDL-C) ≥2.85 mmol/L, serum homocysteine <10 μmol/L, and estimated glomerular filtration rate (eGFR) <90 ml/min/1.73 m^2^, as well as those without smoking. Further analysis of EPVS distribution, the TyG index was found to be associated with moderate to severe EPVS in the centrum semiovale (CSO), not in the basal ganglia (BG). Conclusively, the TyG index was independently and positively associated with moderate to severe CSO EPVS. TyG index may serve as an independent risk factor for CSVD in clinical practice.

## Introduction

Cerebral small vessel disease (CSVD) represents a cluster of pathologies with a heterogeneous etiology, which can affect the brain vascular system, such as small arteries, capillaries, and small veins. CSVD can cause lacunar infarction and cerebral hemorrhage, which accounts for 20–30% of symptomatic strokes (1, 2). Typical CSVD lesions are characterized by white matter hyperintensities of presumed vascular origin, lacunes, microbleeds, enlarged perivascular space (EPVS), and microinfarcts (3). CSVD is recognized as an important cause of cognitive impairment, dementia, increased risk of stroke (4), and worse outcome after stroke (5). It is the most common cause of vascular dementia, contributing to about 50% of dementias worldwide, and, is a massive health burden of stroke and dementia (4). The possible mechanism of CSVD is arteriolosclerosis, lipohyalinosis, or fibrinoid necrosis of small vessels.

Enlarged perivascular space, a neuroimaging hallmark of CSVD, is associated with morphological features of CSVD (3). It is generally most prominent in the inferior basal ganglia (BG) and centrum semiovale (CSO). Epidemiological evidence indicates that the overall EPVS burden is associated with the vascular dementia, Alzheimer's disease, post-stroke depression, and post-stroke cognitive impairment (6, 7). The CSO perivascular space (PVS) develops in middle age and increases with age (8). PVS in the cerebral hemisphere white matter is more common in patients with cerebral amyloid angiopathy (9), while that in the BG is associated with deep perforating artery arteriolosclerosis and subcortical vascular cognitive impairment (10). There are some known risk factors for EPVS, such as old age, hypertension, estimated glomerular filtration rate (eGFR), and hyperuricemia (11). However, other risk factors remain to be further investigated.

Insulin resistance (IR) is a pathological condition resulting from the reduced insulin sensitivity in peripheral tissues (12). Studies have indicated that IR can enhance the activation, adhesion, and aggregation of platelets, and promote atherosclerosis, which may further lead to ischemic stroke (13, 14). Therefore, IR should be accurately measured in clinic and the damage caused by IR should be closely monitored (15, 16). The Homeostatic Model Assessment for Insulin Resistance (HOMA-IR) has been proposed as a surrogate biomarker of IR. However, the HOMA-IR depends on insulin levels that are not usually measured in clinical practice (17).

Insulin resistance can alter the systemic lipid metabolism. Excessive lipolysis can increase the synthesis of hepatic triglyceride (TG) and decrease the levels of high-density lipoprotein (HDL) and the appearance of small dense low-density lipoprotein (LDL) (16, 18). Based on this, the triglyceride-glucose (TyG) index is suggested as a convenient marker for IR (17, 19). Previous studies have confirmed that the TyG index has high correlation with hyperinsulinaemic-euglycaemic clamps (the gold standard technique for assessing IR) and HOMA-IR (20). TyG index has been found to be associated with metabolic syndromes, cardiovascular diseases, cerebrovascular diseases, arterial stiffness, and carotid atherosclerosis (19, 21–24).

An increased TyG index is significantly correlated with both macrovascular diseases and microvascular diseases (17). Many studies that focused on the relationship between IR and CSVD have used HOMA-IR as indicator. For example, HOMA-IR is found to be associated with total CSVD score (25), silent brain infarcts (26), and white matter hyperintensity (WMH) (27), and EPVS (28). However, most of the studies were conducted in a relatively healthy population and did not exclude patients with diabetes. Recently, a cross-sectional study suggested that the TyG index had a slightly stronger association with the prevalence of silent brain infarct and the volume of WMH than HOMA-IR (27). However, little is known about the association of the TyG index with EPVS. If there is a close relationship between TyG index and EPVS, the high-risk group of EPVS will be able to be identified more easily.

Therefore, this study aims to investigate whether the TyG index is associated with EPVS burden independent of other clinical risk factors in non-diabetic adult population. Our findings may provide experimental evidence for further understanding the pathogenesis of EPVS and may provide guidance for early screening, early diagnosis, early prevention, and treatment of CSVD.

## Materials and Methods

### Ethics

This retrospective cross-sectional study was approved by the Institutional Review Board of Quanzhou First Hospital Affiliated to Fujian Medical University (No. [2020]168). The informed consent was waived due to the retrospective design and because only de-identified and anonymized participant information was used. All experiments were performed in accordance with the Declaration of Helsinki and all relevant guidelines and regulations.

### Participants

A total of 531 non-diabetic patients who came to the Department of Inpatient Neurology of the First Affiliated Hospital of Fujian Medical University from January 2017 to December 2020 were enrolled in this study. Inclusion criteria: (1) age between 18 and 85 years. (2) Patients underwent examination with 3.0-T cranial MRI, and the images, such as T1-weighted image (T1WI), T2-weighted image (T2WI), fluid-attenuated inversion recovery (FLAIR), and diffusion-weighted imaging (DWI) were obtained. (3) Patients underwent blood biochemistry, homocysteine, and other related laboratory tests during hospitalization, as well as cranial MRI, head CT scan, and other examinations within 7 days. Exclusion criteria: (1) patients with massive cerebral infarction caused by previous large vessel occlusion, or previous diseases that were not conducive to the discriminant diagnosis of CSVD; (2) patients without MRI examination data; (3) patients with severe heart disease or liver and kidney failure; patients with hematologic diseases or malignant tumors; patients with acute and chronic inflammation; and (4) patients with diseases that may cause ischemic lacunar infarct and white matter damage, such as multiple sclerosis, genetic degenerative diseases, and poisoning.

### Demographic and Clinical Data Collection

Baseline data were collected by the trained study personnel according to standard operating procedures. The contents of the standard questionnaire included age, sex, current medication, previous medical diagnosis, smoking history, and drinking history. Anthropometric parameter measures for the clinical examination included weight, height, systolic blood pressure (SBP), and diastolic blood pressure (DBP). The height and weight of each participant were measured without shoes and heavy clothing. Body mass index (BMI) was calculated as weight (kg)/height (m^2^). Blood pressure was measured using an electronic sphygmomanometer (Omron; Dalian, China), with subjects in a sitting position after 10 min of rest, and the average of three measurements was used. Hypertension was defined when sitting and resting SBP ≥ was 140 mmHg or DBP was ≥90 mmHg at screening, or when patients were taking antihypertensive medications.

### Laboratory Assessment

The fasting blood samples from the participants were obtained at around 7 a.m. and were analyzed at the clinical laboratory department of the Quanzhou First Hospital Affiliated to Fujian Medical University. The blood samples were assessed for the following indicators: white blood cell (WBC), high-sensitivity C-reactive protein (hs-CRP), fasting glucose, total cholesterol (TC), high-density lipoprotein cholesterol (HDL-C), low-density lipoprotein cholesterol (LDL-C), TG, hemoglobin A1c (HbA1c), serum homocysteine, serum uric acid, and creatinine. The TyG index was calculated by the formula of Ln[fasting triglycerides (mg/dl) × fasting glucose (mg/dl)/2] (17, 20). The eGFR was estimated using the Chronic Kidney Disease Epidemiology Collaboration (CKD-EPI) equation.

### MRI Examination

All participants underwent brain MRI using 3.0-T MR scanners (Signa, GE Healthcare, Milwaukee, WI, USA). EPVS was defined as a round, oval, or linear lesion with diameter <3 mm along the course of a perforating artery with signal intensity similar to cerebrospinal fluid in all sequence spaces and without T2 hyperintense rim on FLAIR (3). At the BG and CSO levels, we evaluated the slide in the most affected hemisphere. According to previous description, the EPVS burden was scored as follows: 0 = no EPVS, 1 = 1–10 EPVS, 2 = 11–20 EPVS, 3 = 21–40 EPVS, and 4 = more than 40 EPVS (29). Two neuroradiologists who did not know the clinical details of the patients rated the EPVS. If asymmetric EPVS was observed in the bullous hemisphere, the hemisphere with more EPVSs was selected. Any disagreements regarding EPVS were resolved by consulting a neuroimaging specialist.

### Statistical Analyses

Statistical tests were performed with SPSS 23.0 (IBM SPSS, Chicago, IL, USA) and GraphPad Prism 8 (GraphPad Software Inc., San Diego, CA, USA). We divided the participants into mild (score of 0–1) and moderate to severe (score of 2–4) groups according to the EPVS burden at BG and CSO (29). Continuous variables are presented as median [interquartile range (IQR)], while categorical variables are presented as absolute values and proportions. Since the burden of EPVS was a binary event outcome, we performed univariate analyses using Student's *t*-test or Mann–Whitney *U*-test for continuous variables and the chi-square test or Fisher's exact test for categorical variables. Then, statistically significant (*p* < 0.05 in univariate analysis) and clinically important (sex and BMI) variables were introduced in the multivariate regression analysis. Since the TyG index itself consists of fasting glucose and TG values, fasting glucose and TG were not included as confounders in the multivariate analysis.

The independent association of the TyG index with EPVS was evaluated using the multivariate logistic regression models [odds ratio (OR) and 95% *CI*] with adjustment for major covariables. In addition, possible modifications on the association between TyG index and EPVS were evaluated by stratified analyses and interaction testing. The relationship between the TyG index and vascular risk factors was examined using simple linear regression analyses. The values of *p* < 0.05 were considered statistically significant.

## Results

### Baseline Characteristics

The baseline characteristics of 531 participants were collected and analyzed. The median (Q_L_, Q_U_) age of subjects was 64 (54–71) years. There were 314 (59.13%) men. The median value of the TyG index was 8.45 (8.15–8.82). The baseline characteristics of the groups stratified by EPVS scores are shown in Table 1. The indicators of sex, age, smoking, drinking, SBP, DBP, hypertension, HbA1c, TG, serum homocysteine, creatinine, eGFR, serum uric acid, and hs-CRP were statistically different between moderate to severe EPVS group and mild EPVS group (*p* < 0.05). The group of moderate to severe EPVS had significantly lower levels of HDL-C but significantly higher levels of TyG index than the mild EPVS group (*p* < 0.05).

**Table 1:** Characteristics of subgroups based on the severity of enlarged perivascular space (EPVS).

	**Mild EPVS group (score of 0–1) (*n* = 259)**	**Moderate to severe EPVS (score of 2–4) (*n* = 272)**	***P***
Sex, male, *n* (%)	133 (51.4)	181 (66.5)	<0.001
BMI, kg/m^2^	23.63 [22.03–24.62]	23.61 [21.96–24.81]	0.735
Age, years	58 [49–67]	67 [60–74]	<0.001
Smoking, *n* (%)	62 (23.9)	118 (43.4)	<0.001
Drinking, *n* (%)	29 (11.2)	50 (18.4)	0.020
Hypertension, *n* (%)	95 (36.7)	182 (66.9)	<0.001
SBP, mmHg	132 [117–150]	148.50 [135.00–165.75]	<0.001
DBP, mmHg	84 [75–92]	88.5 [80–98]	<0.001
Fasting glucose, mmol/L	5.06 [4.71–5.54]	5.09 [4.67–5.75]	0.462
HbA1c (%)	5.7 [5.5–5.9]	5.8 [5.6–6.0]	<0.001
Triglyceride, mmol/L	1.13 [0.85–1.49]	1.20 [0.92–1.75]	0.012
TC, mmol/L	4.92 [4.27–5.84]	4.88 [4.07–5.61]	0.117
LDL-C, mmol/L	3.19 [2.63–3.78]	3.09 [2.42–3.72]	0.079
HDL-C, mmol/L	1.21 [1.06–1.43]	1.13 [0.96–1.34]	0.002
Serum homocysteine, μmol/L	8.7 [6.60–12.00]	11.35 [8.53–14.38]	<0.001
Creatinine, μmol/L	64.6 [54.20–78.00]	72.65 [62.37–84.00]	<0.001
eGFR (mL/min per 1.73 m^2^)	99.11 [86.72–108.06]	89.23 [75.54–101.78]	<0.001
Serum uric acid (μmol/L)	322 [269–382]	370.50 [301.75–444.75]	<0.001
WBC (×10^9^/*L*)	6.76 [5.52–8.21]	7.11 [5.78–8.96]	0.090
hs-CRP (mg/L)	1.56 [0.50–2.96]	2.36 [0.54–5.00]	<0.001
TyG index	8.42 [8.11–8.74]	8.51 [8.20–8.93]	0.009

*BMI, body mass index; SBP, systolic blood pressure; DBP, diastolic blood pressure; HbA1c, hemoglobin A1c; TC, total cholesterol; LDL-C, low-density lipoprotein cholesterol; HDL-C, high-density lipoprotein cholesterol; eGFR, estimated glomerular filtration rate; WBC, white blood cell; hs-CRP, high-sensitivity C-reactive protein; TyG, triglyceride glucose*.

### Association of TyG Index With EPVS Burden

A univariate regression analysis showed that age, smoking, drinking, SBP, DBP, serum homocysteine, creatinine, eGFR, serum uric acid, LDL-C, HDL-C, and TyG index were all associated with EPVS burden (Table 2). In the multivariable logistic regression analysis, the TyG index (OR 2.077, 95% CI 1.268–3.403; *p* = 0.004) were independently associated with moderate to severe EPVS (Table 2).

**Table 2:** The logistic regression analysis between possible predictors and severity of EPVS.

	**Univariate analysis**		Multivariable analysis^*^	
	**OR (95% CI)**	***P*-value**	**OR (95% CI)**	***P*-value**
Age	1.064 (1.042–1.085)	<0.001	1.066 (1.042–1.091)	<0.001
Smoking	2.885 (1.866–4.460)	<0.001	2.130 (1.222–3.710)	0.008
Drinking	2.718 (1.563–4.727)	0.021	–	–
SBP	1.024 (1.015–1.032)	<0.001	–	–
DBP	1.034 (1.018–1.050)	<0.001	–	–
Hypertension	3.491 (2.442–4.990)	<0.001	2.068 (1.348–3.172)	0.001
LDL-C	0.905 (0.723–1.132)	0.035	–	–
HDL-C	0.342 (0.162–0.722)	0.006	–	–
Serum homocysteine	1.052 (1.020–1.086)	0.001	–	–
eGFR	0.976 (0.965–0.987)	<0.001	–	–
Serum uric acid	1.006 (1.004–1.008)	<0.001	1.005 (1.002–1.007)	<0.001
TyG index	2.481 (1.577–3.902)	0.001	2.077 (1.268–3.403)	0.004

*SBP, systolic blood pressure; DBP, diastolic blood pressure; LDL-C, low-density lipoprotein cholesterol; HDL-C, high-density lipoprotein cholesterol; eGFR, estimated glomerular filtration rate; TyG, triglyceride glucose*.

* *Adjusted for age, sex, BMI, smoking, drinking, SBP, DBP, hypertension, LDL-C, HDL-C, eGFR, serum uric acid, and serum homocysteine*.

### Subgroup Analyses by Potential Effect Modifiers

**Figure 1:**
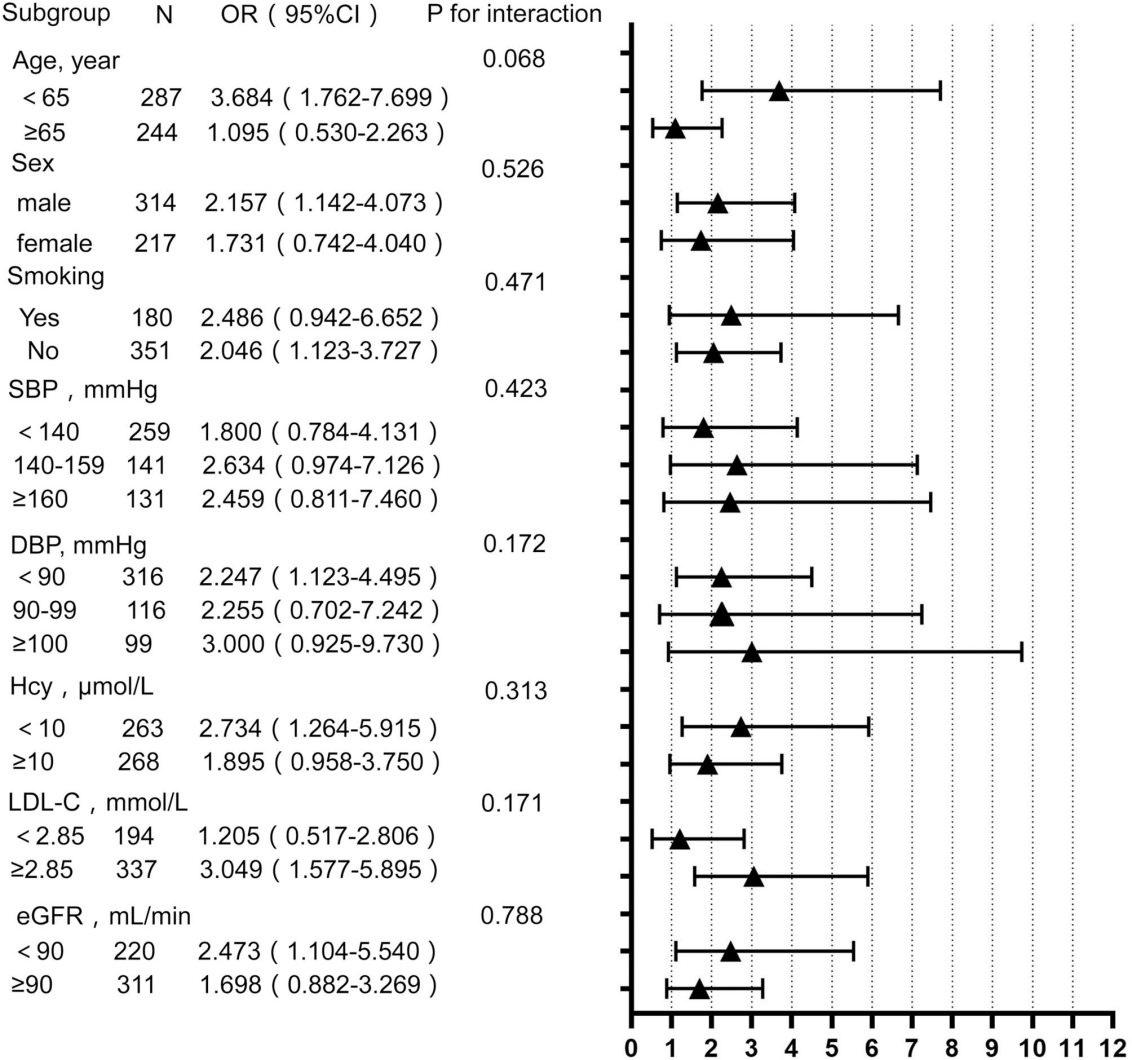
Subgroup analysis for the impact of triglyceride glucose (TyG) index on enlarged perivascular space (EPVS) burden. Each subgroup analysis adjusted, if not stratified, for age, sex, BMI, smoking, drinking, SBP, DBP, hypertension, LDL-C, HDL-C, eGFR, serum uric acid, and serum homocysteine. BMI, body mass index; SBP, systolic blood pressure; DBP, diastolic blood pressure; HDL-C, high-density lipoprotein cholesterol; LDL-C, low-density lipoprotein cholesterol; Hcy, homocysteine; eGFR, estimated glomerular filtration rate.

To determine whether the effect of the TyG index is affected by other factors, the hierarchical analysis and interaction analysis were performed. Figure 1 shows the subgroup analysis of the estimated OR of TyG index for EPVS burden. After adjusting for other confounding variables, the TyG index was significantly associated with an increased risk of moderate to severe EPVS in subgroups of age <65 years (OR 3.684; 95% CI 1.762–7.699), male (OR 2.157; 95% CI 1.142–4.073), DBP <90 mmHg (OR 2.247; 95% CI 1.123–4.495), as well as those without smoking (OR 2.046; 95% CI 1.123–3.727). The same association was observed with LDL-C ≥ 2.85 mmol/L (OR 3.049; 95% CI 1.577–5.895), serum homocysteine <10 μmol/L (OR 2.734; 95% CI 1.264–5.915), and eGFR < 90 ml/min/1.73 m^2^ (OR 2.473; 95% CI 1.104–5.540). However, there was no association between the TyG index and other risk factors.

### Moderate to Severe vs. Mild EPVS in the CSO and BG

Comparison of characteristics between patients with moderate to severe and mild EPVS in the CSO and BG is shown in Table 3. In the CSO, it was found that between patients with different EPVS grades, the TyG index (*p* = 0.004) was significantly different. Significant differences between patients with different EPVS grades were found in sex (*p* = 0.009), age (*p* = 0.014), smoking (*p* = 0.003), hypertension (*p* < 0.001), SBP (*p* < 0.001), DBP (*p* = 0.003), HbA1c (*p* = 0.001), TG (*p* = 0.010), HDL-C (*p* = 0.007), Hcy (*p* = 0.029), creatinine (*p* < 0.001), eGFR (*p* = 0.007), UA (*p* < 0.001), and hs-CRP (*p* = 0.022). However, in the BG, it was found that there was no significant difference in TyG index (*p* = 0.578) between the two groups of EPVS grades, indicating that the TyG index distribution was similar in the moderate to severe BG-EPVS and mild BG-EPVS group.

**Table 3:** Comparison of clinical characteristics between patients with moderate to severe (score of 2–4) and mild (score of 0–1) EPVS in the centrum semiovale (CSO) and the basal ganglia (BG).

	**Mild CSO-EPVS group (score of 0–1)**	**Moderate to severe CSO-EPVS (score of 2–4)**	***P*-value**	**Mild BG-EPVS group (score of 0-1)**	**Moderate to severe BG-EPVS (score of 2–4)**	***P*-value**
No.	332	199		353	178	
Sex, male, *n* (%)	182 (54.8)	132 (66.3)	0.009	187 (53)	127 (71.3)	<0.001
BMI, kg/m^2^	23.62 [22.08–24.61]	23.61 [21.79–24.89]	0.976	23.61 [21.91–24.62]	23.81 [22.66–24.90]	0.026
Age, years	63 [52–71]	64 [58–71]	0.014	60 [51–67]	69 [64–76]	<0.001
Smoking, *n* (%)	97 (29.2)	83 (41.7)	0.003	97 (27.5)	83 (46.6)	<0.001
Drinking, *n* (%)	43 (13.0)	36 (18.1)	0.107	46 (13.0)	33 (18.5)	0.092
Hypertension, *n* (%)	152 (45.8)	125 (62.8)	<0.001	148 (41.9)	129 (72.5)	<0.001
SBP, mmHg	136.5 [122–156]	148 [131–162]	<0.001	136 [120.5–155]	150 [135.75–170]	<0.001
DBP, mmHg	85 [76–95]	89 [79–100]	0.003	85 [75.5–95]	88.5 [80–98]	0.004
Fasting glucose, mmol/L	5.05 [4.66–5.55]	5.1 [4.71–5.78]	0.115	5.09 [4.71–5.56]	5.02 [4.62–5.73]	0.690
HbA1c (%)	5.7 [5.5–5.9]	5.8 [5.6–6.03]	0.001	5.7 [5.5–5.9]	5.9 [5.6–6.1]	<0.001
Triglyceride, mmol/L	1.13 [0.85–1.55]	1.23 [0.93–1.81]	0.010	1.17 [0.87–1.64]	1.16 [0.91–1.66]	0.567
TC, mmol/L	4.89 [4.23–5.73]	4.89 [4.04–5.67]	0.382	4.92 [4.22–5.84]	4.84 [4.04–5.50]	0.069
LDL-C, mmol/L	3.15 [2.59–3.75]	3.04 [2.40–3.73]	0.216	3.19 [2.57–3.78]	3.06 [2.42–3.70]	0.095
HDL-C, mmol/L	1.19 [1.04–1.42]	1.13 [0.95–1.34]	0.007	1.20 [1.05–1.42]	1.10 [0.95–1.34]	0.003
Serum homocysteine, μmol/L	9.35 [7.10–13.2]	10.60 [8.10–13.6]	0.029	9.2 [6.8–12.4]	12 [8.68–16.15]	<0.001
Creatinine, μmol/L	66.40 [55.85–80.70]	91.01 [78.50–102.31]	<0.001	67 [55.9–79.15]	72.85 [62.52–84]	<0.001
eGFR (mL/min per 1.73 m^2^)	97.05 [83.39–106.17]	72 [62–83.2]	0.007	98.31 [84.14–107.01]	88.66 [75.19–101.41]	<0.001
Serum uric acid (μmol/L)	329.5 [272.25–397.25]	372 [306–444]	<0.001	332 [274–395]	376 [304.75–449.5]	<0.001
WBC (×10^9^/L)	6.86 [5.66–8.40]	7.14 [5.74–8.96]	0.222	6.86 [5.51–8.33]	7.14 [5.81–9.06]	0.071
hs-CRP (mg/L)	1.89 [0.50–3.36]	2.30 [0.54–5.0]	0.022	1.76 [0.50–3.30]	2.44 [0.54–5.00]	<0.001
TyG index	8.42 [8.11–8.76]	8.51 [8.22–8.94]	0.004	8.45 [8.14–8.81]	8.45 [8.15–8.88]	0.578

*BMI, body mass index; SBP, systolic blood pressure; DBP, diastolic blood pressure; HbA1c, hemoglobin A1c; TC, total cholesterol; LDL-C, low-density lipoprotein cholesterol; HDL-C, high-density lipoprotein cholesterol; eGFR, estimated glomerular filtration rate; WBC, white blood cell; hs-CRP, high-sensitivity C-reactive protein; TyG, triglyceride glucose*.

The logistic regression analysis of CSO-EPVS revealed that the TyG index, age, sex, SBP, DBP, hypertension, HDL-C, eGFR, and serum uric acid had an association with moderate to severe CSO-EPVS. After adjustment for other confounders, the TyG index (OR 1.936, 95% CI 1.236–3.032; *p* = 0.004) was independently associated with the presence of moderate to severe CSO-EPVS (Table 4).

**Table 4:** The logistic regression of factors associated with CSO-EPVS.

	**Univariate analysis**		Multivariable analysis^*^	
	**OR (95% CI)**	***P*-value**	**OR (95% CI)**	***P*-value**
Age	1.022 (1.006–1.038)	0.008	–	–
Sex	0.616 (0.428–0.887)	0.009	–	–
Smoking	1.733 (1.200–2.504)	0.003	–	–
Drinking	1.484 (0.916–2.405)	0.109	–	–
SBP	1.013 (1.005–1.020)	0.001	–	–
DBP	1.019 (1.006–1.032)	0.004	–	–
Hypertension	2.000 (1.396–2.866)	<0.001	1.467 (0.975–2.206)	0.066
LDL-C	0.882 (0.734–1.061)	0.184	–	–
HDL-C	0.428 (0.227–0.806)	0.009	–	–
Serum homocysteine	1.010 (0.997–1.024)	0.134	–	–
eGFR	0.988 (0.979–0.998)	0.013	–	–
Serum uric acid	1.004 (1.002–1.006)	<0.001	1.004 (1.001–1.006)	0.002
TyG index	1.948 (1.354–2.803)	<0.001	1.936 (1.236–3.032)	0.004

*SBP, systolic blood pressure; DBP, diastolic blood pressure; LDL-C, low-density lipoprotein cholesterol; HDL-C, high-density lipoprotein cholesterol; eGFR, estimated glomerular filtration rate; TyG, triglyceride glucose*.

* *Adjusted for age, sex, BMI, smoking, drinking, SBP, DBP, hypertension, LDL-C, HDL-C, eGFR, serum uric acid, and serum homocysteine*.

### Correlation Between the TyG Index and Other Risk Factors

The results of simple linear regression analyses for the relationship between the TyG index and other risk factors were described in Table 5. The TyG index was significantly correlated with BMI, hypertension, SBP, DBP, LDL-C, HDL-C, creatinine, uric acid, and eGFR, but not WBC and hs-CRP.

**Table 5:** A univariate linear regression analysis between the TyG index and risk factors.

	**β (95%CI)**	***P***
BMI, kg/m^2^	0.043 (0.027 to 0.060)	<0.001
Hypertension	0.161 (0.078 to 0.245)	<0.001
SBP, mmHg	0.003 (0.001 to 0.005)	<0.001
DBP, mmHg	0.006 (0.003 to 0.009)	<0.001
TC, mmol/L	0.141 (0.108 to 0.173)	<0.001
LDL-C, mmol/L	0.150 (0.108 to 0.192)	<0.001
HDL-C, mmol/L	−0.484 (−0.624 to −0.345)	<0.001
WBC (×10^9^/*L*)	0.016 (−0.001 to 0.034)	0.060
hs-CRP (mg/L)	−0.004 (−0.010 to 0.002)	0.215
Creatinine, μmol/L	0.002 (0.001 to 0.004)	0.001
eGFR (mL/min per 1.73 m^2^)	−0.003 (−0.005 to −0.001)	0.005
Uric acid (μmol/L)	0.001 (0.001 to 0.002)	<0.001

*BMI, body mass index; SBP, systolic blood pressure; DBP, diastolic blood pressure; TC, total cholesterol; LDL-C, low-density lipoprotein cholesterol; HDL-C, high-density lipoprotein cholesterol; eGFR, estimated glomerular filtration rate; WBC, white blood cell; hs-CRP, high-sensitivity C-reactive protein; TyG, triglyceride glucose*.

## Discussion

This is the first study to evaluate the relationship between IR and EPVS burden using the TyG index. In 531 patients of our study, 51.22% were observed to have moderate to severe EPVS. After adjusting for possible confounding factors, we found that the TyG index was an independent predictor of moderate to severe EPVS. In addition, our study demonstrated that the association of TyG index with moderate to severe EPVS was more significantly in low age group, male group, normotensive group, and high LDL-C group by stratified analysis. Further analysis of EPVS distribution, the TyG index was found to be associated with moderate to severe EPVS in the CSO, but not in the BG.

Several studies have reported a positive association of IR with CSVD (25–28). Nam et al. conducted a retrospective study on 2,615 Korean subjects and found that the TyG index was strongly associated with CSVD (27). In addition, the TyG index has a better predictive value for IR than HOMA-IR (27). Lee et al. found that IR was independently associated with the presence and severity of silent lacunar infarction in a healthy Korean population (26). Wu et al. found that IR assessed using HOMA-IR was an independent risk factor for EPVS in a Chinese non-diabetic population (28). However, the relationship between IR and EPVS is rarely reported. In our study, we reported an association between the TyG index and EPVS in non-diabetic patients, and this study further refines previous research results.

For the mechanism underlying the association between the TyG index and EPVS, we propose several possible explanations. First, endothelial dysfunction should be considered. Normal endothelium is essential for maintaining glucose and insulin homeostasis in the body, while insulin has a vasodilatory effect on blood vessels (30). When IR occurs in the body, excessive insulin and glucose may enhance the vasoconstrictive effects of insulin and may damage the vessel walls, resulting in a diminished ability of the endothelium to perform the physiological effects of vasodilation, vascular permeability, and maintenance of vascular tension (31, 32). When the above conditions occur in the cerebral vessels, they may cause damage to the blood-brain barrier; and, when the blood-brain barrier is damaged, components within the blood can leak into the PVS, leading to the enlargement of the PVS (33).

Second, inflammatory injury may be involved in the relationship between the TyG index and EPVS. Increased subclinical inflammation and oxidative stress are often found in patients with IR (23, 34). If the vascular endothelium is damaged by inflammation, blood contents may leak into the PVS, and these substances cannot be cleared through the vessel wall, eventually leading to the development of CSVD (35). Our study showed no association between the TyG index and WBC count. However, we found that the distribution of hs-CRP in different grades of EPVS was significantly different in the baseline data (Table 1).

Third, atherosclerosis may be the mechanism of action of IR on EPVS. In previous studies, it has been found that IR is associated with the development of atherosclerosis, and the development and rupture of plaque (13, 14, 18, 23, 34). Our study failed to confirm the relationship between the TyG index and atherosclerosis, but we observed that the TyG index was related to TC and LDL-C (Table 5). Patients with IR are often accompanied by other comorbidities, such as hypertension, diabetes, and low HDL-C levels. The TyG index may reflect their combined impact on the disease.

Finally, the pathogenesis of brain arteriolosclerosis (B-ASC) should also be considered. B-ASC is pathologically characterized mainly by thickening of the vessel wall and morphological changes, with increased tortuosity of small arteries (36). B-ASC is considered to be the underlying cause of WMH (37). In the BG and other regions associated with tortuous vessels, the CSVD markers of WMH, EPVS, and cerebral microbleeds can often be observed. In some cases, these may be related to B-ASC directly. A recent study found that intracranial arteriosclerosis was significantly associated with PVS (38). The pathological changes of intracranial arteriosclerosis are similar to B-ASC, which may be correlated with EPVS. In a quantitative ultrastructural study of blood vessels, it was found that the intracranial capillary basement membrane was markedly thickened in patients with diabetes (39). IR is the pathological basis of diabetes and is likely to be involved in the capillary wall thickening due to diabetes. Therefore, we cannot neglect the role of B-ASC between IR and EPVS.

There was a correlation between TyG index and EPVS distributed in the CSO, but not in the BG. However, the mechanism for this result is unclear. The association of CSO-EPVS with cerebral amyloid angiopathy is possibly due to β-amyloid deposition in the perivascular space, which cannot be cleared (40). We suppose that IR may primarily affect cerebral vessels close to the cortical areas, while have insignificant effects on the deep cerebral vessels. Further studies are needed to validate this speculation.

This study has several strengths. First, we used the TyG index to investigate the association between IR and EPVS burden, which has been reported to be more strongly associated with CSVD than HOMA-IR in a previous study (27). Similarly, the TyG index is more strongly associated with various adverse metabolic states than HOMA-IR (41). Second, the stratified regression analysis and subcomponent layer analysis were performed in this study to further validate the TyG index as an independent risk factor for moderate to severe EPVS. Third, our study was conducted in a non-diabetic population, thus excluding the influence of confounding factors, such as diabetes and the use of hypoglycemic agents.

There are some limitations in this study. First, this study was a retrospective observational study. Second, we excluded subjects with diabetes or taking hypoglycemic drugs, which may lead to the selection bias. However, these individuals represented only about 3.4% of the total enrolled population. Therefore, we believe that this is acceptable when interpreting our main results. Third, we used the TyG index to measure IR instead of using the hyperinsulinaemic-euglycaemic clamps, which is the gold standard in quantifying the insulin sensitivity. Because glucose clamps are expensive, time-consuming, and labor-intensive, they are not suitable for clinical and epidemiological studies. The TyG index is a more facile and currently recognized alternative (20). Fourth, because our study was a cross-sectional study, the causality could not be determined. Fifth, there was no correction for multiple independent tests, which may affect the robustness of our findings. Finally, we did not collect other imaging markers of CSVD, such as WMH, lacunae, cortical superficial siderosis, and cerebral microbleeds in this study. These markers may appear simultaneously in the same patient, and the relationship between IR and EPVS may be disturbed. Further prospective studies are needed to validate our findings.

In summary, a high TyG index was associated with a higher prevalence of moderate to severe EPVS in the non-diabetic population, independent of other clinical risk factors. Our findings suggest that the TyG index is an independent risk factor for EPVS.

## Data Availability Statement

The raw data supporting the conclusions of this article will be made available by the authors, without undue reservation.

## Ethics Statement

The studies involving human participants were reviewed and approved by Institutional Review Board of Quanzhou First Hospital Affiliated to Fujian Medical University (No. [2020]168). Written informed consent for participation was not required for this study in accordance with the national legislation and the institutional requirements.

## Author Contributions

YC analyzed the data and wrote the paper. BC collected and analyzed the data. XZ and MX collected the data. XW provided the guidance for the manuscript and statistical analysis. JC conceived the study and revised the study. All authors have read and approved the submission of the manuscript.

## Funding

This work was supported by grants from the Natural Science Foundation of Fujian Province (Nos. 2020J011284 and 2021J011407) and the Science and Technology Project of Quanzhou, Fujian (No. 2019C030R).

## Conflict of Interest

The authors declare that the research was conducted in the absence of any commercial or financial relationships that could be construed as a potential conflict of interest.

## Publisher's Note

All claims expressed in this article are solely those of the authors and do not necessarily represent those of their affiliated organizations, or those of the publisher, the editors and the reviewers. Any product that may be evaluated in this article, or claim that may be made by its manufacturer, is not guaranteed or endorsed by the publisher.
